# Direct Recycling of Nd–Fe–B Magnets Based on the Recovery of Nd_2_Fe_14_B Grains by Acid‐free Electrochemical Etching

**DOI:** 10.1002/cssc.201902342

**Published:** 2019-10-17

**Authors:** Xuan Xu, Saso Sturm, Zoran Samardzija, Janja Vidmar, Janez Scancar, Kristina Zuzek Rozman

**Affiliations:** ^1^ Department for Nanostructured Materials Jožef Stefan Institute Jamova 39 SI-1000 Ljubljana Slovenia; ^2^ Jožef Stefan International Postgraduate School Jamova 39 SI-1000 Ljubljana Slovenia; ^3^ Department of Environmental Sciences Jožef Stefan Institute Jamova 39 SI-1000 Ljubljana Slovenia

**Keywords:** electrochemistry, etching, extraction, magnets, rare-earth elements

## Abstract

Recycling of end‐of‐life Nd–Fe–B magnets is an important strategy for reducing the environmental dangers associated with rare‐earth mining and overcoming the supply risks associated with the rare‐earth elements. In this study, a novel concept for recycling of sintered Nd–Fe–B magnets by directly recovering the matrix Nd_2_Fe_14_B grains is presented. The procedure is based on the anodic etching of sintered Nd–Fe–B magnets in a nonaqueous dimethylformamide (DMF)/0.3 mol L^−1^ FeCl_2_ bath. Selective recovery of Nd_2_Fe_14_B grains was realized within the applied current density <5 mA cm^−2^ based on the etching priority of phases: metallic Nd > intergranular NdFe_4_B_4_ > matrix Nd_2_Fe_14_B. The total energy consumption of the proposed recycling route is estimated to be 2.99 kWh kg^−1^, which is comparable to the state‐of‐the‐art methods. However, the proposed recycling route is currently the only procedure that enables repeated recycling of sintered Nd–Fe–B magnets in a closed‐loop system.

## Graphical abstract

**Here comes the grain again**: Selective extraction of Nd_2_Fe_14_B grains from sintered Nd–Fe–B magnets has been achieved electrochemically by the preferential etching of their surrounding rare‐earth element‐rich grain boundaries. Recycling of sintered Nd–Fe–B magnets based on the Nd_2_Fe_14_B grain recovery represents a circular economy with low energy footprint.

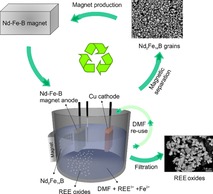

Neodymium–iron–boron (Nd–Fe–B) permanent magnets (PMs) are widely used in many applications, such as hard‐disk drives, wind turbines, acoustic transducers, and electric vehicle drivetrains, owing to their combination of high remanence and high coercivity.^1^ As they incorporate 30–35 wt % rare‐earth elements (REEs), mainly Nd, with small additions of Dy and/or Tb to increase the coercivity and temperature stability, they represent an important secondary resource of REEs.^2^ The total recycling potential for end‐of‐life (EoL) Nd–Fe–B magnets in the period 2016–2040 is estimated to be 233 kt.^3^ As critical materials,^4^ less than 1 % of REEs are being recycled from EoL products, with REE‐containing PMs representing the largest share of these products.^5^ Thus, the recycling of Nd–Fe–B PMs from EoL products is now categorized as a “key enabling technology”^6^ with the prospect of positioning REEs within the circular economy.

The recycling of Nd–Fe–B PMs can be classified into: i) direct re‐use methods, ii) pyrometallurgical processing, and iii) hydrometallurgical separation and recovery.^1^, ^7^ In terms of new magnets production by using recycled EoL products, pyrometallurgical processing working at high temperature is energy‐intensive, whereas hydrometallurgical routes require multi‐processing steps with a large amount of chemical consumption and wastewater generation. In contrast, direct re‐use methods such as resintering,^8^ and hydrogenation disproportionation and desorption and recombination (HDDR)^9^ of EoL sintered Nd–Fe–B PMs are generally regarded as the most economical and ecological ways because they provide short processing steps with zero waste generation. However, the high oxygen content (typically 2000–5000 ppm) in the REE‐rich grain boundary phases of Nd–Fe–B EoL magnets severely limits their recycling potential.^1^, ^10^ These REE oxides (mainly Nd_2_O_3_) cannot be extracted, resulting in reprocessed sintered magnets, lacking full density and exhibiting poor magnetic properties. Therefore, extra REE hydrides are generally added to compensate for the existing REE oxides.8b, 11 This then represents only a partial circular economy for the magnets.^12^ Additionally, the REE‐rich phases, for example, REE oxides, are nonferromagnetic.^13^ With the repeated recycling by direct re‐use methods, the total volume of the nonferromagnetic phases increases owing to the addition of REE hydrides, which then reduces the saturation magnetization and therefore the remanence of sintered Nd–Fe–B magnets. Consequently, sintered Nd–Fe–B magnets produced from the repeated recycling of materials by direct re‐use methods tend to have poorer magnetic properties as the number of cycles increases.

Sintered Nd–Fe–B PMs consist of REE‐rich grain boundaries, representing about 10–12 % of the magnet, and the Nd_2_Fe_14_B grains, which is practically oxygen‐free, accounting for 85–87 % of the magnet.^14^ Thus, direct recovery of the Nd_2_Fe_14_B grains, leaving REE oxides behind as a starting point would provide a sustainable recycling route for fresh Nd–Fe–B PMs production with high magnetic properties.

Herein, we describe an electrochemical process to recover the Nd_2_Fe_14_B matrix grains from sintered Nd–Fe–B magnets based on the etching priority of different phases in the magnets. As a result, the Nd_2_Fe_14_B matrix grains and the REE oxides were disconnected from each other after electrochemical etching, which allowed magnetic separation of the matrix Nd_2_Fe_14_B grains.

To initiate the etching study, the microstructure and the crystal phases of the initial sintered Nd–Fe–B magnets were first investigated (see the Supporting Information, Figure S1). The initial sintered Nd–Fe–B magnet exhibited a typical microstructure that consists of the (Nd_1−*x*_Dy_*x*_)_2_Fe_14_B matrix phase, labeled as “Nd_2_Fe_14_B” for simplicity, surrounded by the REE‐rich grain boundary phases, which mostly consists of metallic Nd and a mixture of different Nd‐based oxides.^15^ The NdFe_4_B_4_ and a mixture of Nd_2_O_3_ and Dy_2_O_3_ phases sitting in some of the triple points are also observed. The electrochemical etching preference of different phases in the Nd–Fe–B magnet was then studied by linear sweep voltammetry (LSV, Figure 1).

**Figure 1:**
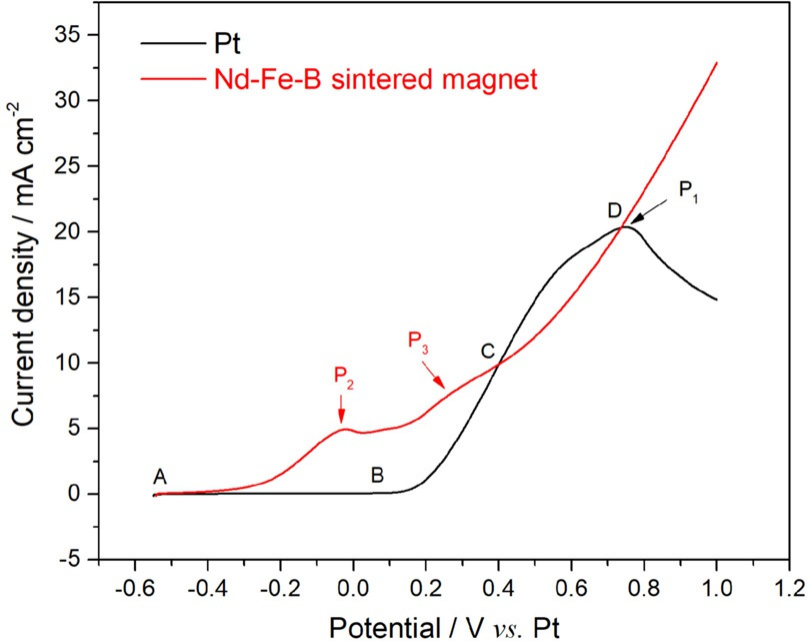
Linear sweep voltammetry of a Pt wire working electrode (black curve) and the initial sintered Nd–Fe–B magnet (red curve) in DMF containing 0.3 mol L^−1^ FeCl_2_, at 40 mV s^−1^, room temperature.

All the possible anodic reactions at the Nd–Fe–B magnet anode are given by Equations (1)–(1):

(1) $$Fe^{2+}-e^{-}\to Fe^{3+},\;\phi_{Fe^{3+}/Fe^{2+}}=-0.41\;V\;\mathrm{vs}.\;SHE$$

(2) $$Nd^{0}-3e^{-}\to Nd^{3+},\;\phi_{Nd^{3+}/Nd^{0}}=-2.32\;V\;\mathrm{vs}.\;SHE$$

(3) $$NdFe_{4}B_{4}-23e^{-}\to Nd^{3+}+4\;Fe^{2+}+4\;B^{3+}$$

(4) $$Nd_{2}Fe_{14}B-37e^{-}\to 2\;Nd^{3+}+14\;Fe^{2+}+B^{3+}$$

When using Pt as the working electrode (black curve), the current density started to increase at approximately 0.15 V along the BC line owing to the onset of the oxidation of Fe^2+^ (reaction 1), which includes also the oxidation of the [FeCl_3_(DMF)]^−[16]^ complex and might explain the mild current peak at approximately 0.55 V and the peak current (P_1_) attributed to [FeCl_4_]^2−[16]^ oxidation at the potential of 0.75 V. When the Nd–Fe–B magnet was used as the working electrode, the current density started to increase at the potential of −0.40 V, shown by the red curve. The peak (P_2_) of 5 mA cm^−2^ recorded at −0.02 V was related to the oxidation of metallic Nd in the grain boundaries (reaction 2) owing to its very negative electrode potential (−2.32 V vs. standard hydrogen electrode, SHE).^17^ The peak (P_3_) at 0.30 V is likely the result of the combined oxidation of the NdFe_4_B_4_ phase (reaction 3) with oxidation of Fe^2+^ (reaction 1). From point C on, the current density regularly increases along CD on the red curve, which is the response of the oxidation of all the Nd‐containing phases together with the Fe^2+^ oxidation (reactions 1–4). Accordingly, the etching priority of the phases inside the magnet is as follows: metallic Nd (in the grain boundary) > NdFe_4_B_4_ > Nd_2_Fe_14_B (magnetic phase). This is in good agreement with previous reports.^18^

Based on the etching priority, selective etching of the metallic Nd from the grain boundary could be realized by applying a potential of <−0.02 V (corresponding to <5 mA cm^−2^) on the anode, whereas applying potentials of higher than 0.40 V (corresponding to >9.9 mA cm^−2^) would lead to the nonselective etching of all the phases present, for example, the metallic Nd phase, the NdFe_4_B_4_, and Nd_2_Fe_14_B (Figure S2).

To reduce the etching of the Nd_2_Fe_14_B grains, a low current density of 2 mA cm^−2^ was applied to study the etching effect on the microstructure of a polished Nd–Fe–B magnet surface (Figure 2 a). The metallic Nd in the roundish (labeled by the white circle), triangular (labeled by the yellow dashed triangle) white phase and the grain boundaries were etched away first (Figure 2 b). The grain boundaries surrounding the Nd_2_Fe_14_B grains were further etched away when the etching time was extended to 2 min (labeled by the yellow dashes, Figure 2 c). When the polished Nd–Fe–B magnet was etched for 4 min, the grain boundaries were completely etched away, exposing the Nd_2_Fe_14_B grains and leaving behind the Dy/Nd‐based oxides (the white phase in the triple points of Figure 2 d), which are not prone to electrochemical oxidation. The gaps (labeled by the white dashes, Figure 2 d,e) between the Nd_2_Fe_14_B grains (the thickness of the gaps varies from approximately 500 nm to 1 μm) were formed after preferential etching of the metallic Nd in the grain boundaries, indicating that some etching of the Nd_2_Fe_14_B grain edges also occurred. The edges of the Nd_2_Fe_14_B grains were further etched perpendicular to the polished surface with a prolonged etching time of 8 min (white arrows, Figure 2 e). When the Nd–Fe–B magnet was etched for 15 min (Figure 2 f), the polished surface was progressively etched, resulting in a porous structure for the Nd_2_Fe_14_B grains and some detachment of the Nd_2_Fe_14_B grains (the position labeled by a white circle). The second layer of the Nd_2_Fe_14_B grains (labeled with white arrows) was much less damaged with the grain boundary completely etched. This indicates that timely removal of the Nd_2_Fe_14_B grains from the magnet body can reduce the further etching the Nd_2_Fe_14_B grains.

**Figure 2:**
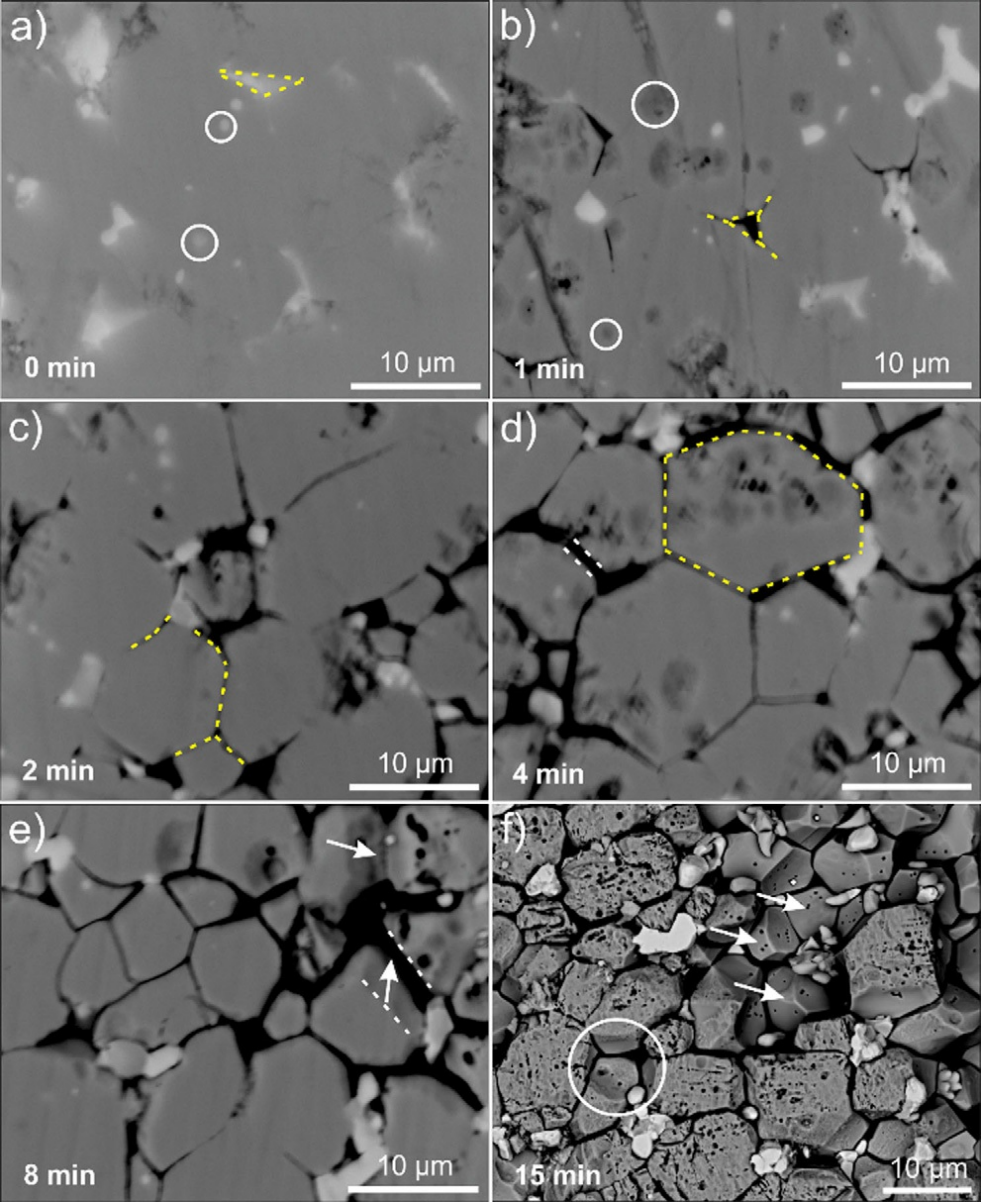
Backscattered electron (BSE)‐SEM images of sintered Nd–Fe–B magnets after electrochemical etching with a current density of 2 mA cm^−2^ for (a) 0, (b) 1, (c) 2, (d) 4, (e) 8, and (f) 15 min at room temperature.

The sintered Nd–Fe–B magnet was electrochemically etched with an applied current of 10 mA (current density of 2 mA cm^−2^) for 360 min to recover the Nd_2_Fe_14_B grains. The magnetically collected particles shown in Figure 3 a are individual particles, confirming that Nd_2_Fe_14_B grains can be extracted through selective etching. X‐ray diffraction (XRD; Figure S3) confirms that these magnetic particles maintain the Nd_2_Fe_14_B crystal structure, which can be re‐used for making new PMs. For the 1.61 g of the sintered Nd–Fe–B magnet treated at 10 mA (2 mA cm^−2^) for 40 h, 1.08 g of Nd_2_Fe_14_B grains were obtained. Accordingly, 67.2 % of the Nd–Fe–B magnet was recovered in the form of Nd_2_Fe_14_B grains and the energy consumption per kilogram of the obtained Nd_2_Fe_14_B grains was calculated to be 0.58 kWh. Around 20 % of the Nd_2_Fe_14_B grains was etched and dissolved into the electrolyte (assuming that the initial Nd–Fe–B magnet contained 87 % Nd_2_Fe_14_B grains).^14^ This is caused by i) the decreasing over‐potential for etching the metallic Nd during the etching process, which forces the etching of the Nd_2_Fe_14_B grains according to the etching mechanism (Figure S4) and ii) untimely removal of the Nd_2_Fe_14_B grains from the magnet anode after the complete etching of the surrounding grain boundaries. However, the recovery of the Nd_2_Fe_14_B grains can be further improved by using an ultrasonic bath during the electrochemical etching process to remove the Nd_2_Fe_14_B grains from the magnet anode in time. The nonmagnetic particles collected by filtering the electrolyte after electrolytic etching are presented in Figure 3 b. The round particles consist of Nd_2_O_3_ and Dy_2_O_3_ phases, whereas the elongated ribbed particles consist of Nd_2_O_3_, Dy_2_O_3_, Nd, and NdB_4_ phases, as confirmed by the energy‐dispersive X‐ray spectroscopy (EDS) and XRD analysis (Figure S5).

**Figure 3:**
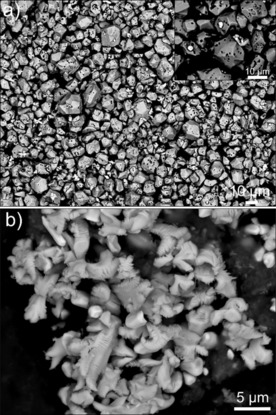
BSE‐SEM images of (a) collected magnetic powder after electrochemical etching (360 min) and (b) collected nonmagnetic particles by filtration after the electrochemical etching (360 min). Etching conditions: 10 mA (2 mA cm^−2^), room temperature, no stirring.

In parallel, pure Fe metal was deposited on the cathode with the current efficiency of 99.6 % (Figure S6 a). As Fe^2+^ was consumed (deposited) on the cathode, while Fe^2+^ and REE ions (REE^3+^), for example, Nd^3+^ were generated from the partly etched magnet anode, the concentration of Fe^2+^, as a whole, decreased in the electrolyte with increasing etching time. In contrast, the concentrations of REE^3+^ in the electrolyte increased linearly with the increasing etching time (Figure S6 b). Therefore, the whole electrolysis process, including the magnet etching on the anode and the Fe deposition on the cathode, ends up with the Nd_2_Fe_14_B grains, REE‐containing electrolyte and REE‐based particles, and pure Fe metal as the final products with only the consumption of FeCl_2_ and electricity.

A recycling route for EoL Nd–Fe–B magnets is proposed based on the electrochemical etching (Figure 4). The obtained Nd_2_Fe_14_B grains are used as raw materials for making new magnets. The REE‐containing electrolyte and REE‐based particles can be further treated by the conventional hydrometallurgical process for a high purity of >99 % REE recovery^1^, 7b followed by molten salt electrolysis^20^ for making RE metals/alloys, which can be used together with the obtained Nd_2_Fe_14_B grains to make new Nd–Fe–B magnets. DMF can be recovered by distillation and re‐used in a closed‐loop with minimized safety risk and environmental impact. Based on that, the overall REE mass balance from the initial magnet is 100 % preserved, which forms a circular economy. The total energy consumption of the magnet‐manufacturing process using the proposed electrochemical recycling route is estimated to be 2.99 kWh kg^−1^, which is directly comparable to the re‐use methods (Table 1), if we consider the conventional additive of the Nd‐Pr hydride (4 wt %). However, the additive can be replaced by other alloys, such as Nd‐Cu^21^ and Ce,^22^ which could lead to a much lower energy footprint.

**Figure 4:**
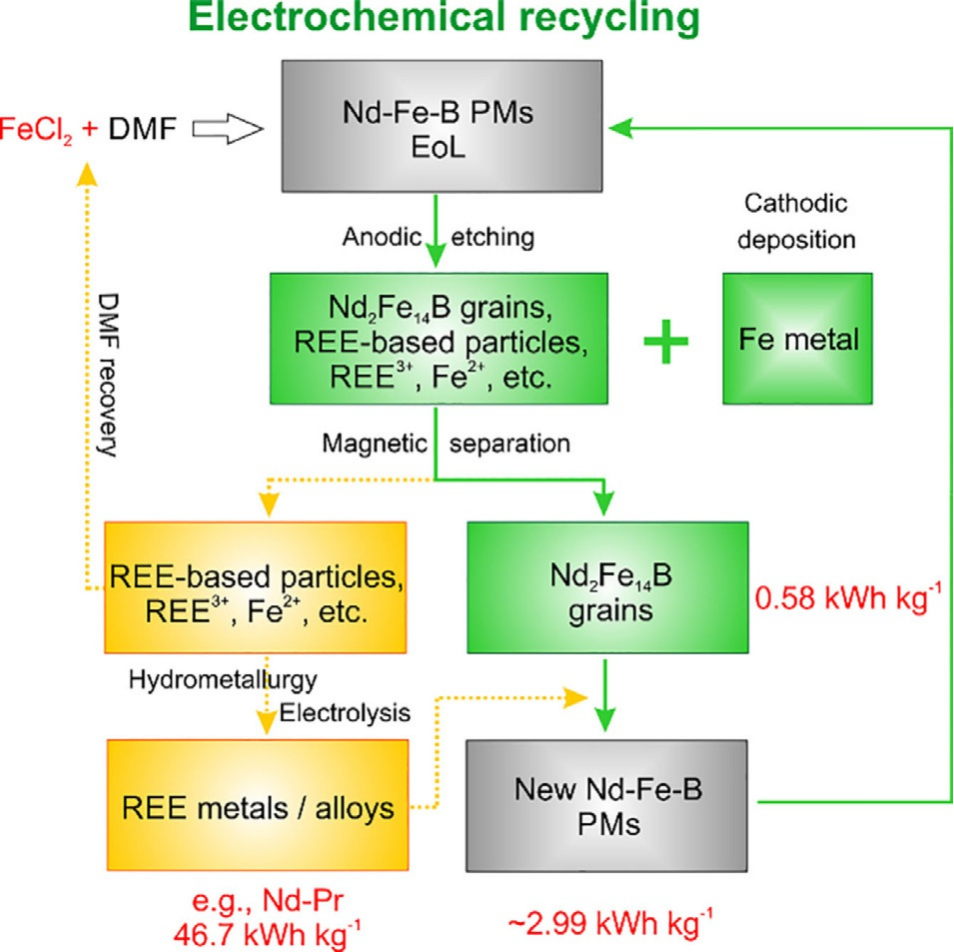
Flowsheet of electrochemical recycling of sintered Nd–Fe–B magnets.

**Table 1:** Energy consumption of making sintered Nd–Fe–B magnets through different recycling routes.^[a]^

Route	Energy consumption [kWh kg^−1^]	Ref.
hydrometallurgy	30.0–33.4	19
direct re‐use	≈3.0	19
electrochemical recycling	≈2.99	this study

[a] The detailed calculation of energy consumption based on previous reports is given in the Supporting Information.

In summary, we are proposing a facile and cost‐effective electrochemical recycling process that selectively recovers the Nd_2_Fe_14_B grains from sintered Nd–Fe–B magnets at room temperature. The anodic etching mechanism is based on fine‐tuning of the applied current density <5 mA cm^−2^ to exploit the etching priority series of the phases present in the pristine Nd–Fe–B magnet: metallic Nd > intergranular NdFe_4_B_4_ > matrix Nd_2_Fe_14_B, which allows the preferential etching of their surrounding REE‐rich grain boundaries, leaving the individual Nd_2_Fe_14_B grains behind for magnetic separation. The total energy consumption of the proposed electrochemical recycling route is estimated to be 2.99 kWh kg^−1^, which is, in the long term, expected to be economically more feasible while offering considerably more flexibility.

## Conflict of interest

*Xuan Xu, Saso Sturm, Kristina Zuzek Rozman, and Jozef Stefan Institute have filed a patent on the presented results. All other authors declare no competing financial interests*.

## Supporting information

As a service to our authors and readers, this journal provides supporting information supplied by the authors. Such materials are peer reviewed and may be re‐organized for online delivery, but are not copy‐edited or typeset. Technical support issues arising from supporting information (other than missing files) should be addressed to the authors.

Supplementary
