# Supplementary material for: Direct Recycling of Nd–Fe–B Magnets Based on the Recovery of Nd2Fe14B Grains by Acid‐free Electrochemical Etching
Source: ChemSusChem. 2019 Oct 17;12(21):4754–8. doi: 10.1002/cssc.201902342 (PMC6916163; doi:10.1002/cssc.201902342)
Supplement: Supplementary file 1 — Supplementary [file CSSC-12-4754-s001.pdf]

CHEMISTRY & SUSTAINABILITY
CHEMSUSCHEM
ENERGY & MATERIALS

Supporting Information

Direct Recycling of Nd–Fe–B Magnets Based on the Recovery of $Nd_2Fe_{14}B$ Grains by Acid-free Electrochemical Etching

Xuan Xu,*[a, b] Saso Sturm,[a, b] Zoran Samardzija,[a] Janja Vidmar,[c] Janez Scancar,[b, c] and Kristina Zuzek Rozman[a, b]

cssc_201902342_sm_miscellaneous_information.pdf

Author Contributions

J.S. Formal analysis: Supporting; Writing - Review & Editing: Supporting.

Supporting Information
©Wiley-VCH 2016
69451 Weinheim, Germany

A novel concept for direct Nd–Fe–B recycling based on the $Nd_2Fe_{14}B$ grains recovery via acid-free electrochemical etching

Xuan Xu,*[a, b] Saso Sturm,[a, b] Zoran Samardzija,[a] Janja Vidmar,[c] Janez Scancar,[b, c] and Kristina Zuzek Rozman[a, b]

Abstract: Recycling of end-of-life Nd–Fe–B magnets is an important strategy for reducing the environmental dangers associated with rare-earth mining and overcoming the supply risks associated with the rare-earth elements. In this study, a novel concept for recycling of sintered Nd–Fe–B magnets by directly recovering the matrix $Nd_2Fe_{14}B$ grains is presented. The procedure is based on the anodic etching of sintered Nd–Fe–B magnets in a non-aqueous dimethylformamide (DMF)-0.3 mol L^{-1} $FeCl_2$ bath. Selective recovery of $Nd_2Fe_{14}B$ grains was realized within the applied current density < 5 mA cm^{-2} based on the etching priority of phases: metallic Nd > intergranular $NdFe_4B_4$ > matrix $Nd_2Fe_{14}B$. The total energy consumption of the proposed recycling route is estimated to be ~2.99 kWh kg^{-1}, that is comparable to the state-of-the-art methods. However, the proposed recycling route is currently the only procedure that enables a repeated recycling of sintered Nd–Fe–B magnets in a closed loop system.

DOI: 10.1002/anie.2016XXXXX

Table of Contents

1. Experimental Procedures
   1.1. Materials and Chemicals
   1.2. Linear sweep voltammetry (LSV)
   1.3. Electrochemical etching of Nd–Fe–B magnets
   1.4. Particles collection
   1.5. Characterization
2. Results and Discussion
   2.1. Microstructure and crystal phases investigation of the initial sintered Nd–Fe–B magnets
   2.2. Electrochemical etching of sintered Nd–Fe–B magnets
   2.3. XRD of the collected magnetic particles
   2.4. The electrochemical etching mechanism of sintered Nd–Fe–B magnets
   2.5. EDS and XRD of the collected non-magnetic particles
   2.6. REE^{3+} & Fe^{2+} concentration in the electrolyte and Fe deposition on the cathode
   2.7. Calculation of energy consumption
3. References

1. Experimental Procedures

1.1 *Materials and Chemicals*: As sintered Nd-Fe-B magnets are quite sensitive to oxygen and water, in order to prevent the oxidation of the recovered $Nd_2Fe_{14}B$ grains that can readily be oxidized, a protective Ar atmosphere for material handling and non-aqueous solvents for the electrochemical experiments need to be used. To select the most suitable solvent, important points need to be considered: i) the solvent should not be based on water, because of the oxidation issues, ii) it should have high solubility for $FeCl_2$ to have a high conductivity of the electrolyte; ii) it should have high vapor pressure, so it can be recovered by distillation and re-used. After considering these facts, the economic feasibility together with environmental and safety issues have to be considered. For this study dimethylformamide (DMF, >99%, Sigma-Aldrich, Germany) was selected for all the electrochemical experiments as it fulfills the first three criteria. Here we have to emphasize precautions have to be met when working with this solvent according to safety regulations.[1] Prior to use, molecular sieves (4A, Sigma-Aldrich, China) that were dried under vacuum at 160 ℃ for more than 24 hours were added to the DMF to remove any water. $FeCl_2{\cdot}4H_2O$ (>99.99%, Sigma-Aldrich) was dehydrated under vacuum at 140 ℃ for 24 hours. All the dried chemicals were stored inside a closed bottle in an argon-filled glove box with water and oxygen contents below 1 ppm. The water concentration in the electrolyte was less than 50 ppm, as determined with a Karl Fischer titration (C20S, Mettler-Toledo, Switzerland) in the electrolyte was less than 50 ppm. The sintered Nd–Fe–B magnets were supplied by Magneti Ljubljana d.d., Slovenia and were used as model materials of EoL PMs. These magnets were demagnetized and mechanically polished to remove the protective coating on the surface (Al/Al_2O_3). The chemical compositions of the magnet scrap were measured with inductively coupled plasma optical emission spectrometry (ICP-OES, Perkin Elmer Optima 5300 DV). Briefly, 0.25 g of the crushed magnets was completely dissolved in a mixture solution of 65% HNO_3 and 37% HCl with volume ratio: 3:1 for 24 h at 23 ± 1 ℃. Then the solution was diluted to a convenient volume using 0.5 mol L^{-1} HNO_3. The composition of the prepared solution was measured using ICP-OES and recalculated into weight percentages of the elements in the magnet with the results listed in Table S1.

Table S1. Elemental composition of Nd–Fe–B magnets in wt.%.

Element	Content (wt.%)	Element	Content (wt.%)
Nd	26.01	Ga	0.22
Dy	7.20	Al	0.12
Fe	61.83	Cu	0.11
Pr	0.58	Co	2.93
B	0.9	Total	99.90

1.2 *Linear sweep voltammetry (LSV)*: A three-electrode cell with a potentiostat (Gamry, Reference 600, USA) was used for all the electrochemical measurements. The electrochemical behaviours of the Nd–Fe–B magnets in 15 mL of DMF containing 0.3 mol L^{-1} $FeCl_2$ at room temperature were evaluated using linear sweep voltammetry (LSV). A polished Nd–Fe–B magnet (2 mm × 2 mm × 30 mm) and a Pt wire (ϕ = 0.5 mm) were used as the working electrode with effective areas of 0.05 and 0.047 cm^2, respectively. Another Pt wire (ϕ = 0.5 mm) was used as the quasi-reference electrode (QRE). All the potentials reported herein were referred to the Pt QRE. A Pt plate (30 mm × 10 mm) was selected as the counter electrode, with an effective area of 2 cm^2.

1.3 *Electrochemical etching of Nd–Fe–B magnets*: A Cu foil substrate (40 mm × 30 mm) was employed as the cathode with an effective area of 10 cm^2 and a polished Nd–Fe–B magnet (2 mm × 5 mm × 30 mm) was used as the anode with the effective area of 0.25 cm^2. A Pt wire (ϕ = 0.5 mm) was used the QRE. The applied current density was within the range 2–48 mA cm^{-2} for various times. In order to monitor the concentration of Nd^{3+} and Dy^{3+} and obtain a higher yield of particles, a Cu-foil substrate (40 mm × 30 mm) was employed as the cathode with an effective area of 10 cm^2 and a polished Nd–Fe–B magnet (2 mm × 15 mm × 30 mm) was used as the anode with an effective area of 5 cm^2. A Pt wire (ϕ = 0.5 mm) was used as the quasi-reference electrode. A current of 10 mA (the corresponding anode current density was 2 mA cm^{-2} and the cathode current density was 1 mA cm^{-2}) was applied for 360 min. A longer etching time of 40 h under the same conditions was applied to collect more particles for energy consumption calculation. A few studies have reported that REEs and transition metals (TMs) could be co-deposited in organic solvents.[2] In order to obtain REE-free metal on the cathode, the cathode current density in current study was controlled at 1 mA cm^{-2}, because the literature[3] suggests that a Nd–Fe film would be obtained at a cathode current density > 1 mA cm^{-2}. All the experimental procedures were carried out in an Ar atmosphere at room temperature.

1.4 *Particles collection*: Since the magnetic particles formed after the etching tended to be attracted by the anode, after every 60 min the etched anode was put close to an external magnet to separate these particles from the anode. After the electrochemical etching, the magnetic particles are collected magnetically and washed three times ultrasonically using DMF. The non-magnetic particles were collected by filtering the electrolyte after etching.

1.5 *Characterization*: The electrolyte was measured by mass spectrometry with inductively coupled plasma (ICP-MS, Agilent 7700, Agilent Technologies, Tokyo, Japan). The collected magnetic and non-magnetic particles were characterized using a scanning electron microscope (SEM, JSM-7600F and 5800, JEOL, Japan) equipped with an energy-dispersive X-ray spectrometer (EDS). An accelerating voltage of 18 kV was used with a working distance of 15 mm for the imaging and the EDS analysis in JSM-7600F. In order to achieve good accuracy for the quantitative elemental analysis, the efficiency of the EDS detector was calibrated at 18 kV using a pure Co-standard. For the JSM-5800, an accelerating voltage of 20 kV was used with a working distance of 10 mm for the imaging and the EDS analysis. The crystal structures of the collected magnetic and non-magnetic particles were determined with an X-ray diffractometer (XRD, PANalytical, Netherlands) using Cu–$K_{\alpha 1}$ radiation (λ = 1.5406 Å).

2. Results and Discussion

2.1 Microstructure and crystal phases investigation of the initial sintered Nd–Fe–B magnets

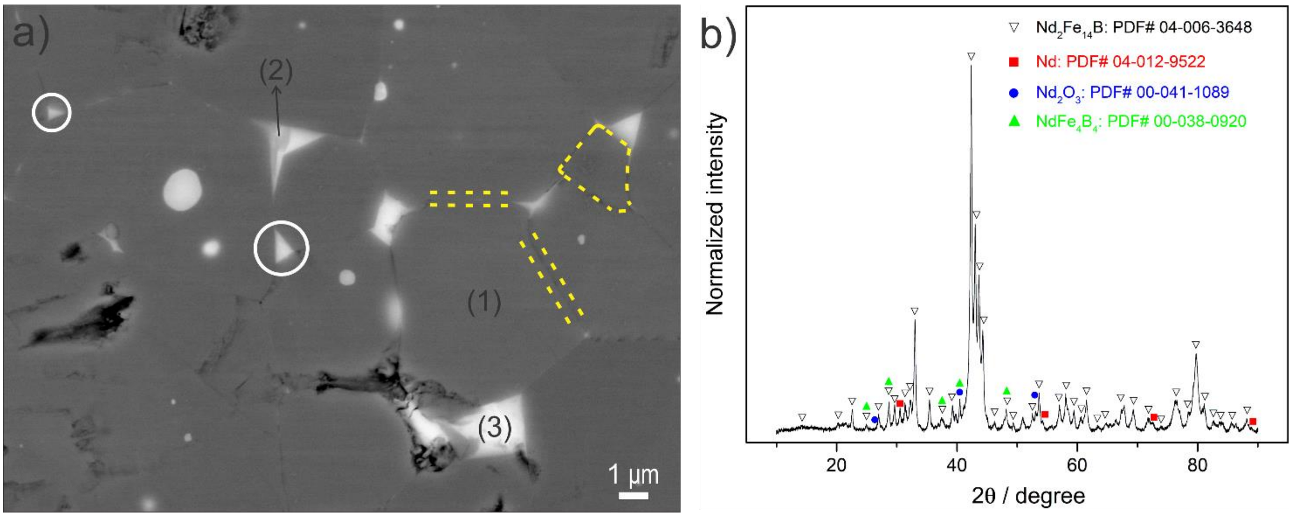

Figure S1. (a) BSE-SEM image and (b) XRD pattern of sintered Nd–Fe–B magnet.

The microstructure of the sintered Nd–Fe–B magnets was first investigated using scanning electron microscopy (SEM). The back-scattered electron (BSE) SEM image (Figure S1a) shows a typical microstructure of the polished Nd–Fe–B magnet that consists of the

dark-grey phase, the matrix phase, labelled (1), containing 83.51 at.% Fe, 9.02 at.% Nd, 3.99 at.% Dy and 3.48 at.% Co, the light-grey phase, labelled (2), containing 63.58 at.% Fe, 30.02 at.% Nd, 2.38 at.% Dy and 4.02 at% Pr, and the white phase, labelled (3), containing 30.23 at.% Nd, 3.89 at.% Dy and 65.88 at.% O. The bright intergranular region surrounding the grey phase (1) are the grain-boundary phases (labelled with yellow dashed lines in Figure S1a). The observed phases are in accordance with literature reports, with the thickness of the grain boundaries being less than 10 nm.[4] The $Nd_2Fe_{14}B$ (PDF# 04-006-3648), metallic Nd (PDF# 04-012-9522) and Nd_2O_3 (PDF# 00-041-1089) phases are observed from X-ray diffraction (XRD) pattern of Nd–Fe–B magnet. Combining the XRD result with the microstructure, the grey phase (1) is the $(Nd_{1-x}Dy_x)_2Fe_{14}B$ matrix phase, i.e., labelled as "$Nd_2Fe_{14}B$" for simplicity. Based on the stoichiometry from energy-dispersive X-ray spectroscopy (EDS) analysis, the light-grey phase (2) is the $NdFe_4B_4$,[5] the peaks of which are overlapping with the peaks of $Nd_2Fe_{14}B$ phase in the XRD pattern, because it represents just a few percent of the total (Figure S1b). The white phase (3) is a mixture of Nd_2O_3 and Dy_2O_3 phases, where the characteristic peaks of the Dy_2O_3 phase are not visible due to there being only a small amount. The analysis of the grain boundary and the triple point (Figure S1a, labelled with white circles) is, however, not reliable in our case, because of its small volume. According to the literature,[4b,6] the grain-boundary phase is REE-rich material, which mostly consists of metallic Nd and a mixture of different Nd-based oxides.

2.2 Electrochemical etching of sintered Nd–Fe–B magnets

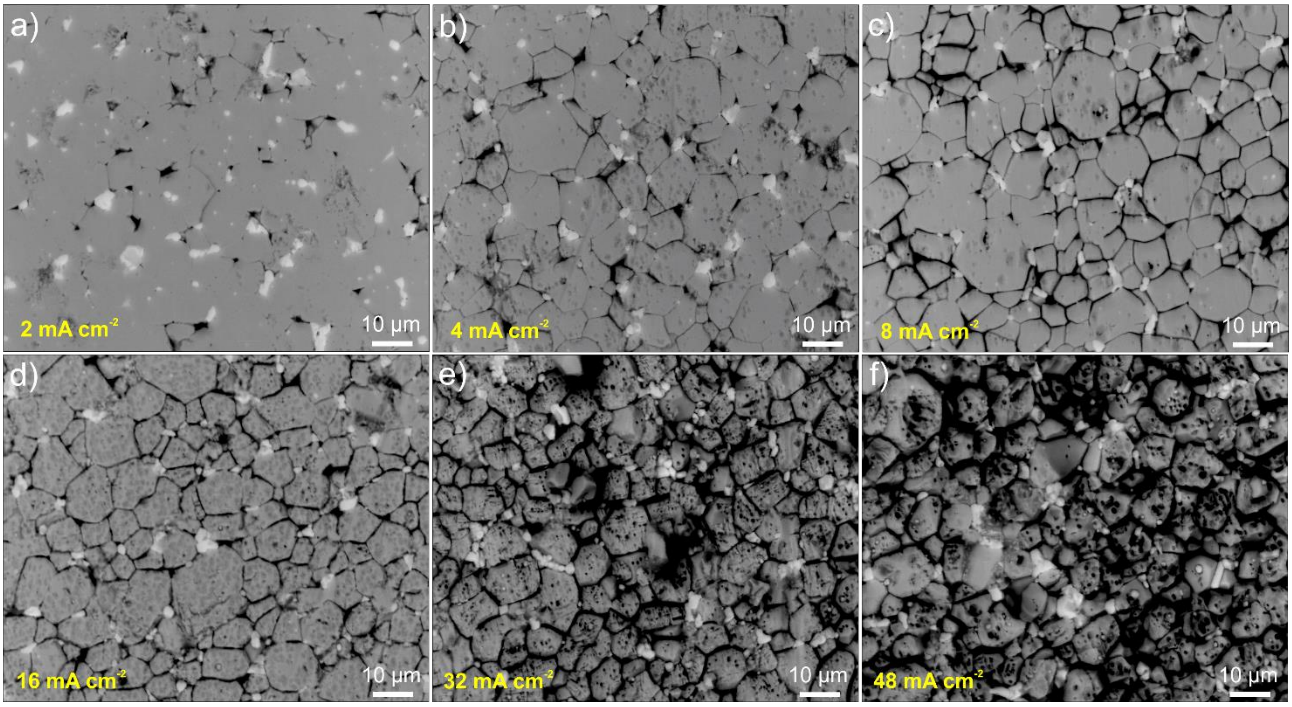

Figure S2. BSE-SEM images of sintered Nd–Fe–B magnets after electrochemical etching with a current density of (a) 2 mA cm^{-2} (b) 4 mA cm^{-2}, (c) 8 mA cm^{-2}, (d) 16 mA cm^{-2}, (e) 32 mA cm^{-2}, and (f) 48 mA cm^{-2} for 2 min at room temperature.

The BSE-SEM images of sintered Nd–Fe–B magnets after electrochemical etching with a current density of 2–48 mA cm^{-2} for 2 min at room temperature are shown in Figure S2. The Nd-rich triple points are preferentially etched at 2 mA cm^{-2} due to the more negative electrochemical potential caused by the higher Nd content (Figure S2a). The Nd-rich grain boundaries connected with the triple points were also slightly etched. A longer etching time is needed to completely etch away the grain boundaries surrounded the $Nd_2Fe_{14}B$ grains. Whereas the grain boundaries were further etched with the increasing applied current density of 4 mA cm^{-2} (Figure S2b) and were completely etched at 8 mA cm^{-2} (Figure S2c) within 2 min, exposing the single $Nd_2Fe_{14}B$ grains on the polished magnet surface. Clearly, the average thickness of the gaps between the $Nd_2Fe_{14}B$ grains was roughly ~100 nm and ~300 nm with the etching current density of 4 and 8 mA cm^{-2}, respectively. As the thickness of the grain boundary of the Nd–Fe–B magnet is generally < 10 nm,[3] the much larger thickness of the gaps between the $Nd_2Fe_{14}B$ grains after etching at 4 and 8 mA cm^{-2} for 2 min indicates that the etching of the edges of the $Nd_2Fe_{14}B$ grains happened together with the etching of the grain boundaries. When the applied current density was further increased from 16 to 48 mA cm^{-2}, aggressive etching on the $Nd_2Fe_{14}B$ grains is clearly observed (Figure S2d–f). Dense craters on the surface of the $Nd_2Fe_{14}B$ grains were formed by etching at 16 mA cm^{-2} (Figure S2d). When the current density of 32 mA cm^{-2} was applied, the morphology (Figure S2e) consists of electrochemically inert Nd/Dy oxides (white phase) and porous $Nd_2Fe_{14}B$ grains in which an average diameter of the pores is roughly 500 nm. A more porous structure of the $Nd_2Fe_{14}B$ grains with an average diameter of the pores around 1 µm was observed under the applied current density of 48 mA cm^{-2} (Figure S2f). Therefore, it can be concluded that for a constant etching time, a higher applied current density results in more aggressive etching of the $Nd_2Fe_{14}B$ grains. A low

current density, e.g., 2 mA cm^{-2} could be applied with long etching time to completely etch the grain boundaries and consequently to obtain the $Nd_2Fe_{14}B$ grains.

2.3 XRD of the collected magnetic particles

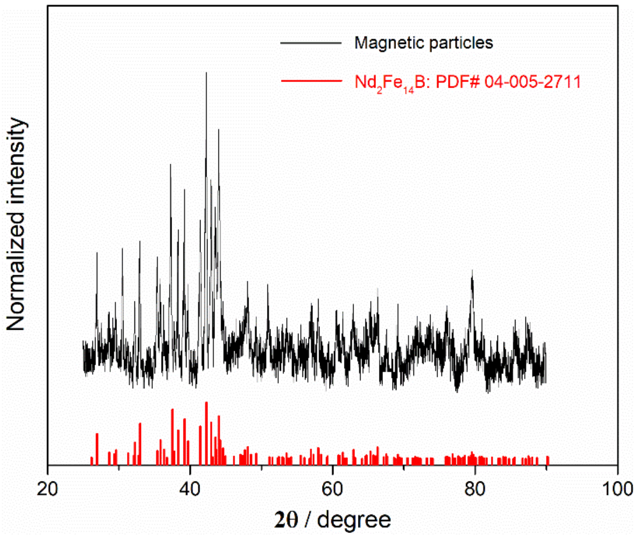

Figure S3. XRD pattern of the collected magnetic particles. Reflection characteristic of magnetic particles matches with $Nd_2Fe_{14}B$ phase (Reference PDF# 04-005-2711).

2.4 The electrochemical etching mechanism of sintered Nd–Fe–B magnets

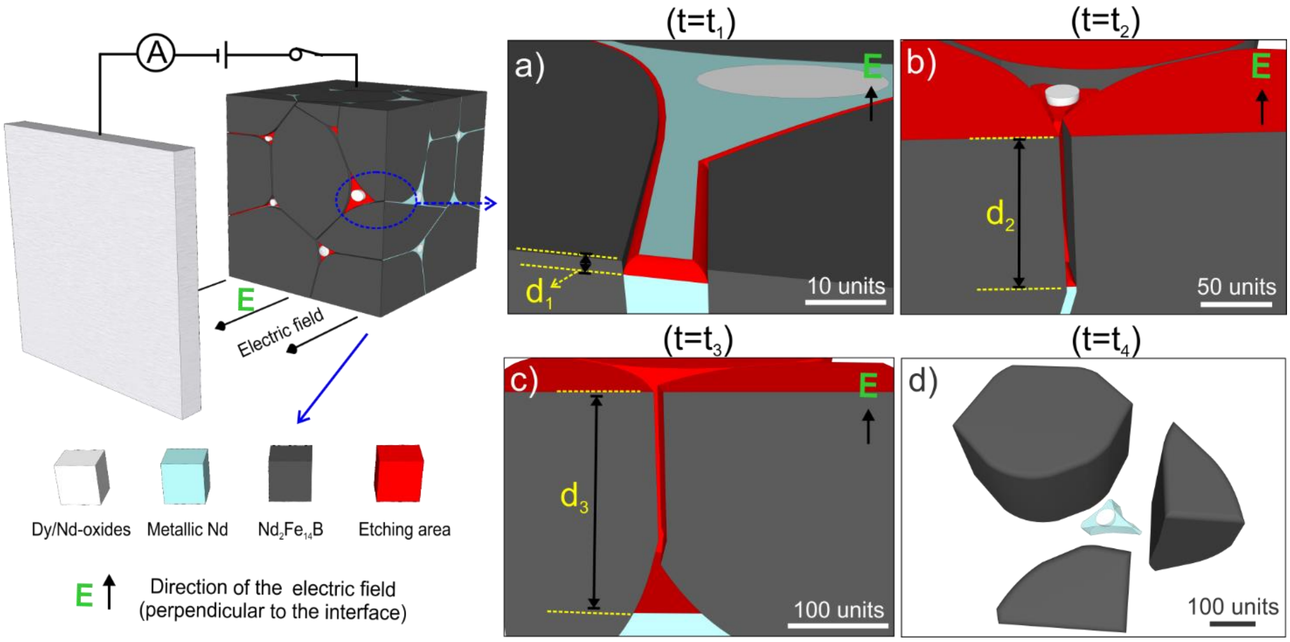

Figure S4. Schematic illustration of the microstructural evolution of sintered Nd–Fe–B magnet with metallic Nd in the grain-boundary phase during electrochemical etching/anodization in a water- and oxygen-free organic electrolyte.

In order to better understand the etching process in three-dimensional space, a mechanism for the electrochemical etching of the sintered Nd–Fe–B magnet with metallic Nd at both the grain boundaries and triple points in a water- and oxygen-free organic electrolyte is illustrated in Figure S4. For simplicity, only the planar surface of the magnet directly facing the cathode is considered to be subject to the electrochemical etching and the low current density (< 2 mA cm^{-2}) is considered to selectively etch the sintered magnets. At the

very beginning $t = t_1$, the anodization begins with the etching of metallic Nd, most probably at the interfaces of the Nd-rich phase and the $Nd_2Fe_{14}B$ grains, as the electrochemical potential is always higher at the electrode edges.[7] As shown in Figure S4a at $t = t_1$ (red coloured regions), an etching depth of d_1 is reached. Etching of the magnet proceeds until $t = t_2$, when a certain thickness (d_2) of the metallic Nd has been etched away, now exposing the $Nd_2Fe_{14}B$ grains (Figure S4b). At $t = t_2$, the metallic Nd in the triple points might not be etched completely, depending on its volume at the triple point, as shown in Figure S4b. The etching depth d for $t \rightarrow t_2$ increases. Consequently, the exposed area of the anode/magnet becomes larger with $t \rightarrow t_2$, which decreases the current density and the over-potential for etching the metallic Nd. As a result, the etching rate of the metallic Nd in the grain boundary reduces with $t \rightarrow t_2$. When a constant current is applied and to keep the charge balanced, the etching of the $Nd_2Fe_{14}B$ grains needs to occur at d_2 at $t = t_2$ (Figure S4b, red-coloured region). At $t = t_3$, the etching reaches a depth of d_3, at which point the etching of the $Nd_2Fe_{14}B$ grains proceeds both on the front interface and the side interfaces (Figure S4c, red-coloured region) in parallel with the deeper etching of the Nd-rich grain boundaries. When the latter is completely etched ($t = t_4$), the partially etched $Nd_2Fe_{14}B$ grains and the Dy/Nd-oxides detach from the magnet surface (Figure S4d).

2.5 EDS and XRD of the collected non-magnetic particles

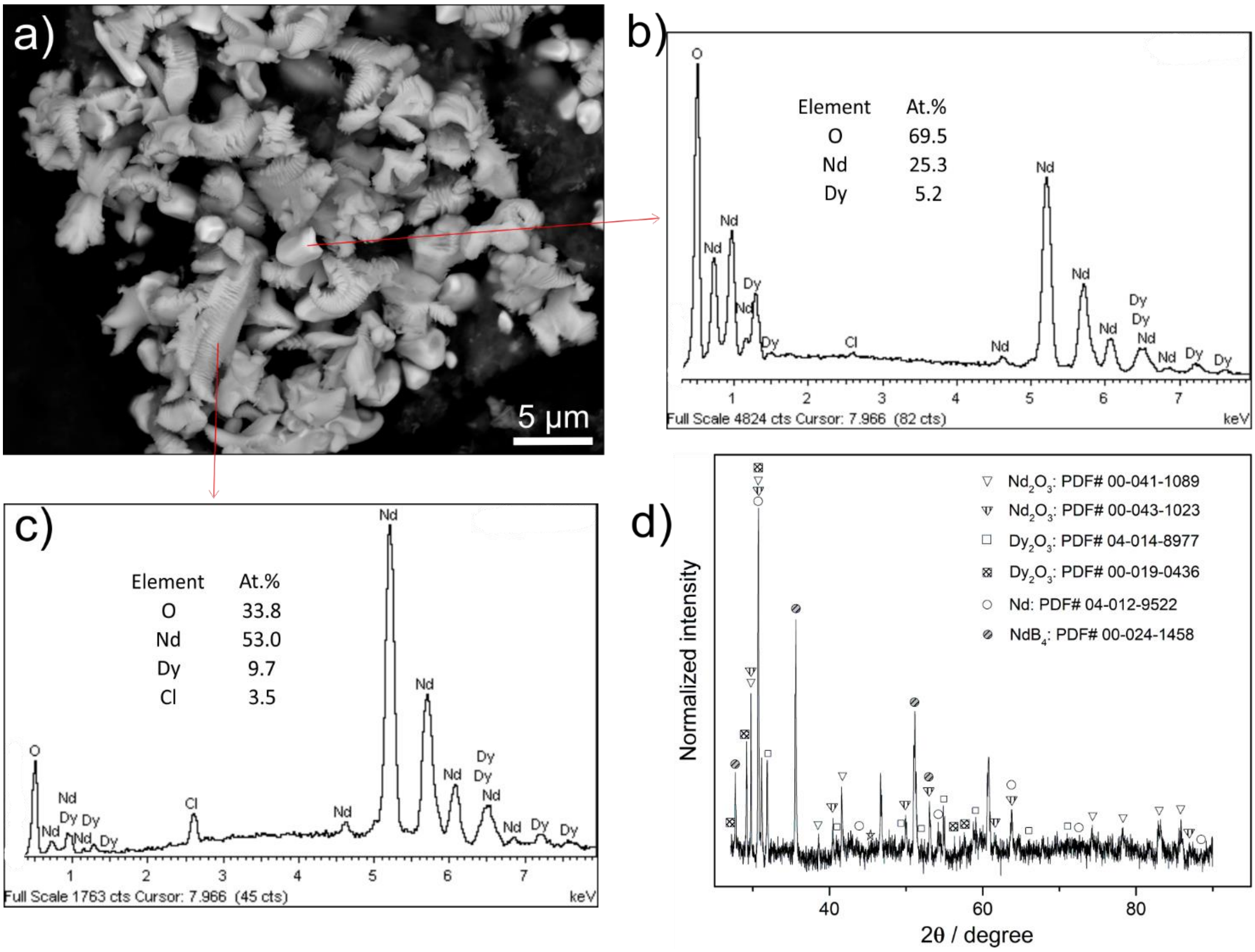

Figure S5. (a) BSE-SEM image of the collected non-magnetic particles obtained by filtration after electrochemical etching (360 min), (b) EDS spectrum of the round shape particle, (c) EDS spectrum of the elongated ribbed particle and (d) The corresponding XRD pattern of the non-magnetic particles of (a). Etching conditions: 10 mA (2 mA cm^{-2}), room temperature, no stirring.

Figure S5a shows a BSE-SEM image of the collected non-magnetic particles obtained by filtration after electrochemical etching (360 min). The round particles and the elongated ribbed particles mainly consist of Nd, Dy and O, in which the oxygen content (O/(Nd + Dy + O)) is ~ 69 at.% and ~35 at.% (Figure S5b and c). The spectra also show the Cl (< 3.5 at.%) coming from the incomplete washing of the particles. It must be noted that boron (B) is not shown in the EDS spectra due to it being a light element, which cannot be detected by EDS. Figure S5d shows the XRD pattern of the non-magnetic particles obtained by filtration after anodic etching of the Nd–Fe–B magnet. The confirmed phases are Nd_2O_3 (PDF# 00-041-1089 & PDF# 00-043-1023), Dy_2O_3 (PDF# 04-014-8977 & PDF# 00-019-0436), Nd (PDF# 04-012-9522) and NdB_4 (PDF# 00-024-1458). Combined with the EDS results, the round particles contain Nd_2O_3 and Dy_2O_3 phases, while the elongated ribbed particles consist of Nd_2O_3, Dy_2O_3, Nd and NdB_4 phases.

2.6 REE^{3+} & Fe^{2+} concentration in the electrolyte and Fe deposition on the cathode

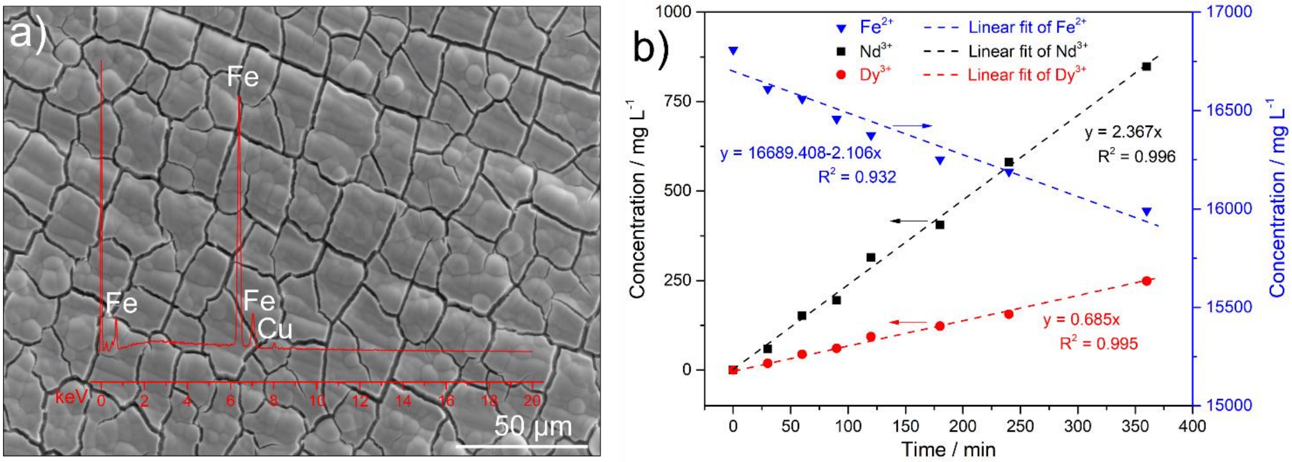

Figure S6. (a) The SE-SEM image of the deposit on the cathode with EDS spectrum (inset). (b) The influence of etching time on the concentrations of Fe^{2+} (blue curve), Nd^{3+} (black curve) and Dy^{3+} (red curve) and in the electrolyte. Conditions: Applied current 10 mA, the corresponding anode current density: 2 mA cm^{-2} and cathode current density: 1 mA cm^{-2}, room temperature, no stirring.

On the cathode, a deposit was obtained after a deposition of 60 min on a Cu substrate/cathode (in the period 301 to 360 min) which is shown in Figure S6a. Some cracks are observed from the secondary-electron SEM image of the deposited film. The EDS spectrum (inset of Figure 6b) indicates that only Fe was deposited on the Cu substrate. This is because the concentration of Fe^{2+} in the electrolyte was much higher than that of Nd^{3+} and Dy^{3+}, and the standard electrode potential of Fe^{2+}/Fe (–0.447 V) is much lower than those of REE^{3+}/REE (< –2.2 V), both of which result in the preferential deposition of Fe. Additionally, the current efficiency of the Fe deposition was 99.6%, because of the use of DMF, which is electrochemically stable, avoiding the hydrogen evolution that generally happens in aqueous solutions.[8] The concentrations of dissolved Nd^{3+}, Dy^{3+} and Fe^{2+} are monitored along with the etching time of 360 min (Figure S6b) at the applied current of 10 mA (the corresponding anode current density is 2 mA cm^{-2}). It can be seen from Figure S6b that the concentrations of Nd^{3+} and Dy^{3+} in the electrolyte increase with the increasing etching time and exhibit a linear relationship (correlation coefficient, R^2 = 0.996 and 0.995, respectively), from which the etching rate of the Nd^{3+} and Dy^{3+} are calculated as 2.367 and 0.685 mg L^{-1} min^{-1}, respectively. The concentration of Fe^{2+}, in contrast, decreases with the increasing etching time, which indicates that the consumption of Fe^{2+} in the electrolyte due to electrodeposition on the cathode was larger than the amount of Fe^{2+} dissolved into the electrolyte by etching from the anode. The net consumption rate of Fe^{2+} is calculated as 2.106 mg L^{-1} min^{-1}. Therefore, the only chemical consumption for recovering the $Nd_2Fe_{14}B$ grains is $FeCl_2$.

2.7 Calculation of energy consumption

To make new Nd–Fe–B magnets using the recovered $Nd_2Fe_{14}B$ grains as the starting material, the addition of REE metals or alloys, e.g., Nd hydride[9] and Nd–Pr hydride[10] are expected to reach fully dense magnets with the formation of grain boundaries. 1.9 wt.% of Nd–Pr hydride/cycle is generally added in direct re-use methods to compensate for the loss of the Nd with the continuous recycling cycles due to Nd_2O_3 formation in the grain boundaries.[9-10] Upon that the energy consumption for producing 1.0 kg of sintered Nd–Fe–B magnets via direct re-use methods is ~3.0 kWh when using the direct re-use methods, with 1.9 wt.% of Nd–Pr hydride additive.[10] The total energy consumption for making new Nd–Fe–B magnets via the proposed electrochemical recycling route therefore includes: i) the energy consumed for recovering the $Nd_2Fe_{14}B$ grains, ii) the energy consumed for additive REE metals or alloys making,[10] here we are considering the double addition of the Nd–Pr hydride (~4 wt.% Nd–Pr hydride) as we have to compensate for the whole Nd-rich grain boundary phase, and, iii) the energy consumed for the new Nd–Fe–B magnet processing steps. The energy consumption per kilogram for making Nd–Pr hydride associated with mining the ores is 46.7 kWh,[10] and the energy consumption per kilogram of the obtained $Nd_2Fe_{14}B$ grains was calculated to be 0.58 kWh. Considering also the magnet processing steps, such as sintering (0.46 kWh kg^{-1}) and annealing (0.08 kWh kg^{-1}),[10] the total energy consumption of the magnet-manufacturing process with blending 1.9 wt.% (0.89 kWh) and 4 wt.% of Nd–Pr hydride (1.87 kWh) using the proposed electrochemical recycling route is estimated to be ~2.01 kWh kg^{-1} and ~2.99 kWh kg^{-1}, respectively. It has to be emphasized that the proposed electrochemical recycling route requires a constant amount of the additive per cycle irrespective of the number recycling cycles as the Nd-rich phase does not pick up oxygen while recycling, unlike in direct re-use methods. Consequently, higher total energy consumption is expected for direct re-use methods with the repeating recycling cycles. For comparison, the energy consumption of the hydrometallurgical route is regarded the same as that of the traditional magnet-manufacturing route associated with mining the ores, due to their similar production process.[11] The total energy consumption for producing 1.0 kg of sintered Nd–Fe–B magnets is 30.0–33.4 kWh using the traditional magnet-manufacturing route.[10] As a whole, the proposed electrochemical recycling route proves itself to be comparable in energy demand to the direct re-use methods, but it proves to be more sustainable in the long term.

References

[1] Sigma-Aldrich, in *Material safety data sheet*, **2004**.

[2] a) L. Peng, Y. Qiqin, Y. Yansheng, T. Yexiang, *J. Rare Earth.* **1999**, *2*; b) J. X. Li, H. Lai, B. Q. Fan, B. Zhuang, L. H. Guan, Z. G. Huang, *J. Alloy. Compd.* **2009**, *477*, 547-551.

[3] N. Yoshimoto, O. Shinoura, H. Miyauchi, M. Ishikawa, Y. Matsuda, *Denki Kagaku Oyoki Kogyo Butsuri Kagaku* **1994**, *62*, 982-984.

[4] a) Y. Shinba, T. Konno, K. Ishikawa, K. Hiraga, M. Sagawa, *J. appl. phys.* **2005**, *97*, 053504; b) L. Yanfeng, Z. Minggang, L. Anhua, F. Haibo, S. HUANG, L. Wei, D. An, Q. Yan, *J. Rare Earth.* **2014**, *32*, 628-632; c) X. Xu, T. Sasaki, Y. Une, H. Kubo, T. Ohkubo, M. Sagawa, K. Hono, *Acta Mater.* **2018**, *151*, 293-300; d) K. Hono, H. Sepehri-Amin, *Scripta Mater.* **2012**, *67*, 530-535.

[5] K. Oesterreicher, H. Oesterreicher, *J. Less Common Met.* **1984**, *104*, 19-21.

[6] S. Wang, Y. Li, *J. Magn. Magn. Mater.* **2005**, *285*, 177-182.

[7] A. J. Bard, L. R. Faulkner, J. Leddy, C. G. Zoski, in *Electrochemical methods: fundamentals and applications, 2nd Edition, Vol. 2*, Wiley New York, **2000**.

[8] J. Zhang, P. Evans, G. Zangari, *J. Magn. Magn. Mater.* **2004**, *283*, 89-94.

[9] O. Diehl, M. Schönfeldt, E. Brouwer, A. Dirks, K. Rachut, J. Gassmann, K. Güth, A. Buckow, R. Gauß, R. Stauber, *J. Sustain. Metall.* **2018**, *4*, 163-175.

[10] M. Zakotnik, C. O. Tudor, L. T. Peiró, P. Afiuny, R. Skomski, G. P. Hatch, *Environ. Technol. Innovation* **2016**, *5*, 117-126.

[11] M. Reimer, H. Schenk-Mathes, M. Hoffmann, T. Elwert, *Metals* **2018**, *8*, 867.
